# Supplementary material for: DySurv: dynamic deep learning model for survival analysis with conditional variational inference
Source: J Am Med Inform Assoc. 2024 Nov 21;33(1):112–22. doi: 10.1093/jamia/ocae271 (PMC12758469; doi:10.1093/jamia/ocae271)
Supplement: ocae271_Supplementary_Data [file ocae271_supplementary_data.pdf]

TABLE I: Features extracted from the SUPPORT survival dataset.

**Feature**	**Type**	**Feature**	**Type**
Sex	binary	Age	integer
Number of comorbidities	integer	SUPPORT Coma Score	continuous
Years of Education	continuous	APS III Score	continuous
Diabetes	binary	Dementia	binary
3rd Day MAP	continuous	3rd Day WBC	continuous
3rd Day HR	continuous	3rd Day RR	continuous
Temperature	continuous	3rd Day Bilirubin	continuous

TABLE II: Features extracted from the NWTCO survival dataset.

**Feature**	**Type**	**Feature**	**Type**
Relapse	binary	Age	integer
Histology Local	binary	Histology Institute	binary
Tumor Stage	categorical	Study Type	categorical

I. Libraries and Packages

Statistical and clinical analyses were performed using R (version 3.6.2, R Foundation for Statistical Computing, Vienna, Austria) with packages including binom, Epi, ggplot2, lme4, sjstats, tableone, and tidyverse. Machine learning components were coded using Python (version 3.8.0) with packages including imblearn, matplotlib, skopt, xgboost, seaborn, shap, pandas, numpy, PyCox, and sklearn.

II. Appendix

A. *Data Description*

A detailed list of features used in the study from different datasets can be seen in Tables I, II, III, IV and V.

TABLE III: Features extracted from the GBSG survival dataset.

Feature	Type	Feature	Type
Hormone Treatment	binary	Age	integer
Tumor Grade	categorical	Menopause Treatment	binary
Progesterone (fmol)	continuous	Number of positive nodes	categorical
		Estrogen (fmol)	continuous

TABLE IV: Features extracted from the METABRIC survival dataset.

Feature	Type	Feature	Type
Age	integer	Gene I	binary
Gene II	binary	Gene III	binary
Hormone Treatment	binary	Gene IV	binary
Radiotherapy	binary	Chemotherapy	binary
ER-positive	binary		

B. Parametric Models

The Cox model is often described as a semi-parametric model due to the exponential nature of the function applied to the predictors when estimating the hazards. Other more recent deep learning models like deep survival machines and deep recurrent survival networks assume *a priori* a specific statistical distribution like the Weibull or a combination of a few similar distributions for the survival distribution ie. the event times. The task then becomes estimating the parameters of these distributions using deep learning architectures. Making such strong parametric assumptions on the nature of the survival distribution, a stochastic distribution, limits the potential predictive capabilities of these models [1]. Parametric survival models estimate these distributional parameters (such as the shape of the Weibull) as a function of the features $\mathcal{X}$. The probability density for an event at time t given these parameters θ is

$$f(t \mid \boldsymbol{\theta}), t \geq 0,$$

$$\boldsymbol{\theta} = \boldsymbol{\theta}(\mathcal{X}) = (\theta_1(\mathcal{X}), \theta_2(\mathcal{X}), \ldots) = \left(g_1\left(\mathcal{X}, \boldsymbol{\beta}_1\right), g_2\left(\mathcal{X}, \boldsymbol{\beta}_2\right), \ldots\right),$$

where $g_1(), g_2(), \ldots$ are real-valued functions associating features $\mathcal{X}$ with the distributional parameters $\boldsymbol{\theta}(\mathcal{X})$ via parameters $\boldsymbol{\beta}_1, \boldsymbol{\beta}_2, \ldots$. These functions can be taken to be neural network modules accordingly. The models then maximise the likelihood given the observed data

$$L(\boldsymbol{\theta}) = \prod_{i=1}^{n} L_i(\boldsymbol{\theta}) = \prod_{o \in \mathcal{O}} f(y_o) \times \prod_{c \in \mathcal{C}} S(y_c) \times \prod_{l \in \mathcal{L}} (1 - S(y_l)) \times \cdots ,$$

C. Benchmark Models

To compare DySurv to other survival analysis methods, we implement a selection of the most popular and consistently cited methods in the field and provide a short description of each below. We will first introduce discrete-time methods which rely on discretising the event times into specified durations, and then follow with continuous-time methods.

1) PMF: The parametrisation of the Probability Mass Function (PMF) of the event times is another way of estimation without resorting to using discrete-time risk or hazard in likelihood optimisation. We described the continuous probability density function without using discrete time boundaries. It is the foundation of other methods like DeepHit and Multi-Task Logistic Regression. It similarly resorts to optimising a negative log-likelihood loss but instead of using the cumulative risk function, it uses the approximations of the PMF and survival functions [2]. Since we can establish a direct representational relation between the risk and survival functions, the PMF loss can be seen as an alternative to our loss function. In our and others' implementation, the PMF method is a simple Multi-Layer Perceptron (MLP) optimised for this loss and we use the same structure as we use for our survival module to be described later.

2) MTLR: Multi-Task Logistic Regression provides a generalization of the binomial log-likelihood to jointly model the sequence of binary labels for each time interval risk prediction.

This method similarly minimises the negative log-likelihood with the PMF and survival function terms but the network outputs are cumulatively summed in reverse to no certain advantage and, in fact, just add computational complexity and numeric instability [3], [4].

3) BCESurv: This Binary Cross-Entropy for Survival is a method consisting of a set of binary classifiers that remove individuals as they are censored. The loss is the binary cross entropy of the survival estimates at a set of discrete times, with targets that are indicators of surviving each time. Each output node in the last layer corresponds to a binary classifier evaluated at that time point. As censored patients are removed, the method is biased towards those with higher event probabilities [5].

4) DeepHit: The single-risk version of DeepHit is a deep learning model whose output nodes are softmaxed to jointly model the probabilities between the event times and the time durations are discretised like in our case. The model depends on optimising both the negative log-likelihood loss based on the cumulative incidence function and a ranking loss built on the intuition of the concordance. The ranking loss penalises incorrect ordering of patient pairs in which the patient that remains longer in the study should have a lower risk at the endpoint for the patient with the shorter stay. Including this loss function allows the model to optimise for concordance. The concordance was the only evaluation metric used in the paper which might have lead to potentially biased and inflated results. Subsequently reproduced work has shown that indeed this model is not calibrated well and significantly lags across other metrics when compared to simpler models [6]–[9].

5) Logistic Hazard: The Logistic Hazard method is a submodular implementation of our deep learning model using the loss in (4) with an MLP that similarly parametrises the PMF of the survival times [10]. DySurv expands on this method to include it as a component in the framework with the variational autoencoder to jointly optimise for both tasks of reconstruction and latent space formation as well as survival estimation. A key difference between the log-likelihood loss used here (and in our model) and in DeepHit is that logistic hazards do not allow for survival past the maximum time horizon.

6) CoxTime: CoxTime is a relative risk model that extends Cox regression beyond the proportional hazards and is the first of the continuous-time methods. The standard Cox regression model which we will not spend space introducing here consists of a baseline hazard term (defined cumulatively in the loss by a pre-selected estimator such as Breslow) and a relative risk term which is an exponential factor of the weighted linear combination of features. The basic model assumes constant proportionality between the patients' hazards over time and is thus restrictive. In other words, the difference between survival likelihoods for a given time is proportional to the difference in feature or hazard values for patients. CoxTime goes around this assumption by parametrising the relative risk term as a function of time and not just the features, thus the non-proportional behaviour over time is modelled by allowing for time to be considered alongside the features [11].

7) CoxCC: CoxCC (Case Control) is just a proportional implementation of the CoxTime model and is the closest to the standard Cox implementation where the minimisation of the partial log-likelihood is done with stochastic gradient descent by averaging over constrained risk sets for mini-batch learning instead of the entire dataset like in classical survival analysis [11].

8) DeepSurv: DeepSurv is a deep learning model that directly minimises the negative partial log-likelihood as defined in the standard Cox model. It is one of the first deep learning implementations for survival analysis and the output of the model is the log-risk term of the Cox model which accounts for nonlinearity [12]. There is no indirect estimation of cumulative risk or survival through likelihood estimation like in the previous methods, thus DeepSurv similarly suffers under limitations of the Cox such as the proportionality assumption [13].

9) PCHazard: The last continuous-time method we introduce and implement is PCHazard which assumes that the continuous-time hazard rate (instantaneous value of risk) is piecewise constant. The method relies on optimising for the likelihood contribution which mimics the MTLR approach albeit in the continuous-time setting with the hazards parametrised by a simple MLP. The piecewise constant causes the likelihood to behave like a Poisson likelihood [14]. Despite the method operating in continuous-time, the hazards are defined in time intervals which rely on discretisation steps from the observed continuous event times and censoring times, while we discretise the times to a predefined set of time flagposts [2].

D. LSTM Cell

Long-Short-Term-Memory units were introduced to solve instability in training encountered with an earlier recurrent architecture, namely recurrent neural networks which kept losing information from earlier in the sequence [15]. A thorough tutorial on the module can be found here: [16]. Long-term dependencies not captured by recurrent neural networks are more efficiently learned by using LSTMs mostly due to their new state in the architecture, the cell state. This

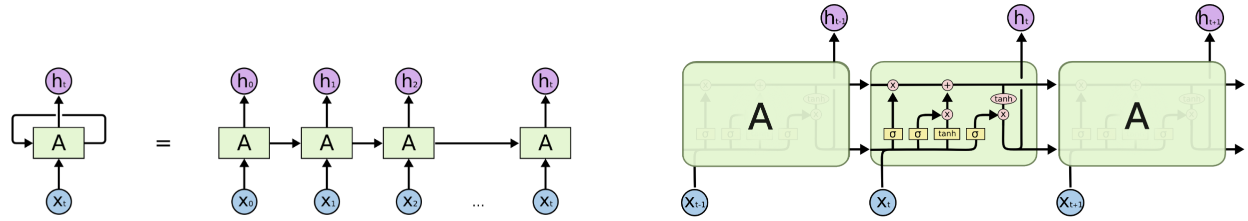

(a) (b)

Fig. 1: The recurrent neural network (RNN) (a) is unrolled to show how the output information, $\mathrm{h_t}$, is a result of previously passed information and the current update from a learning module A. LSTMs (b) have a more complex architecture as the top horizontal line connecting the modules (the cell state) is now passing from the previous to the next time step directly with some potential updates depending on how the gates σ and $tanh$ are activated from the input X [17].

cell state propagates information from previous time points to the next forecasted point largely untouched, thus helping preserve long-term dependency. Occasionally, there is information in the sequence that should be used to update past information, such as a very high measurement of heart rate undetected previously, and that is included in the cell state by passing it through gates. These gates correspond in a way to the neurons of a neural network, if a value is over a certain threshold, then activate the gate and pass on information, and if not, do not do anything to the cell state. An LSTM has three such gates and they help filter what information should be added from the most recent time point to what has been learned before. A comparison of a recurrent neural network and an LSTM can be seen in Figure 1.

E. Model architecture

Following the LSTM unit, the remaining part of the encoder consists of an MLP module with 3 layers. The encoder and decoder are mirrored in their structure with the hidden neurons in the MLP layers consisting of 3 times feature length, then 5 times, then 3 times, before passing

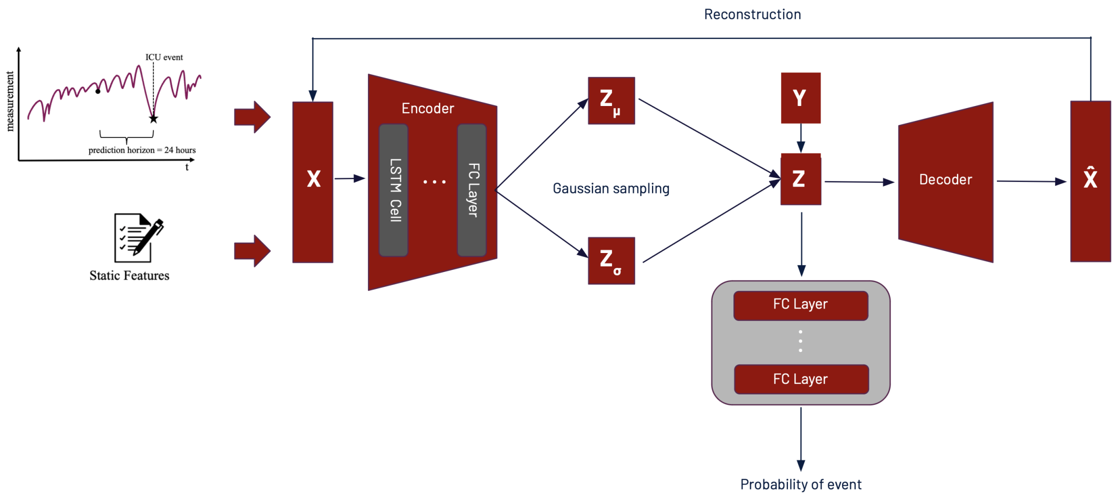

Fig. 2: Proposed DySurv model for dynamic risk prediction based on a combination of static and time-series EHR data

onto the last output layer. For the encoder, the last layer is used in approximating the mean and standard deviation of the latent Gaussian distribution. As for the decoder, the last output layer is used for reconstructing the input vector. The survival module is similar to the MLP components of the encoder and decoder. All components are jointly optimised through multi-loss optimisation. All of the components have been investigated with and without dropout included. The output of the survival module is 10 nodes softmaxed hence properly jointly distributed, each of which gives the probability that the patient has suffered the event (death) at that specific time interval.

F. Ablation studies

To empirically arrive at the optimal configuration for the architecture, we undertook ablation studies and grid search combinations for hyperparameters using a validation set presented in the tables below. The hyperparameters investigated for DySurv include:

- *Dropout*: 0.5, 0.6, 0.7, 0.8

- *Number of hidden layers*: 2, 3, 4, 5
- *Number of hidden neurons*: 10, 25, 75, 100, current
- *Latent representation vector length*: 10, 20, 30
- *Learning rate*: 0.00001, 0.0001, 0.001, 0.01, 0.1
- *Batch size*: 8, 16, 32, 64, 128, 256, 512

G. Interpretability

To investigate whether the model is learning relevant clinical attributes of patient representation for ICU survival, we interpreted the model predictions as a function of the input. By using permutation importance approaches, we iteratively remove each feature and measure the change in concordance on the test set when done so over 10 iterations. We do this for every feature and the amount by which the concordance changes is then a measure of how important that removed feature is to the prediction. In Figure 3, we show the results in our two datasets, MIMIC-IV and eICU. The model identified age and sex, creatinine, O_2 saturation, albumin, platelet count, and blood pressure to be predictive of ICU survival. This is in line with relevant medical research [18]–[21]. The increased platelet count is probably correlated as research suggests with a possible infection that affects patient survival. Research also suggests that exposure to hypotension during the stay in the ICU was associated with increased mortality and associations increased with increasing hypotension severity.

H. Framework Overview

The proposed framework for the paper can be seen in 4 highlighting the interdisciplinary components of the project.

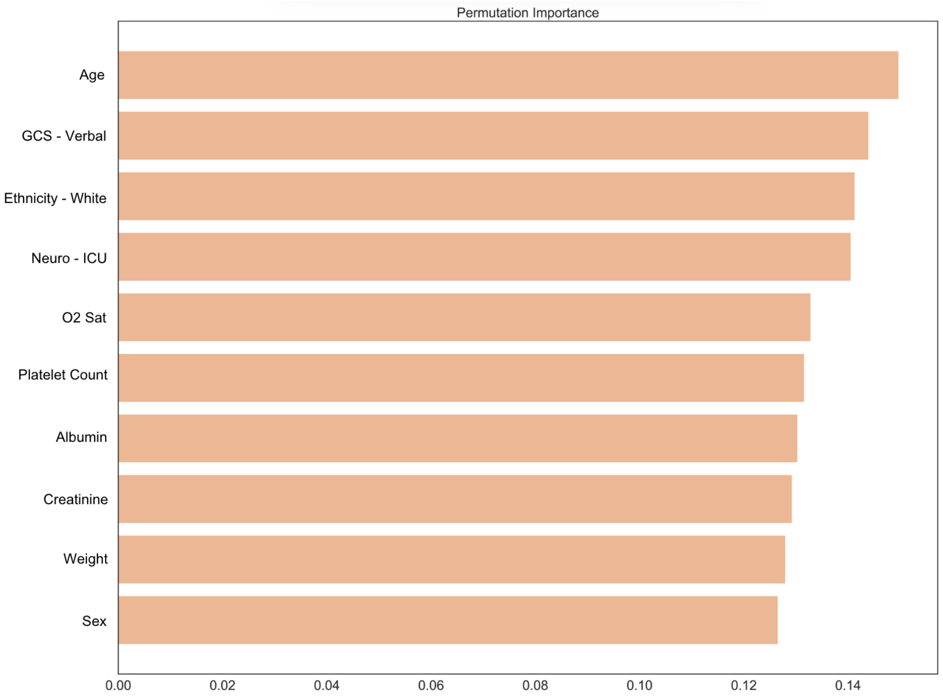

(a)

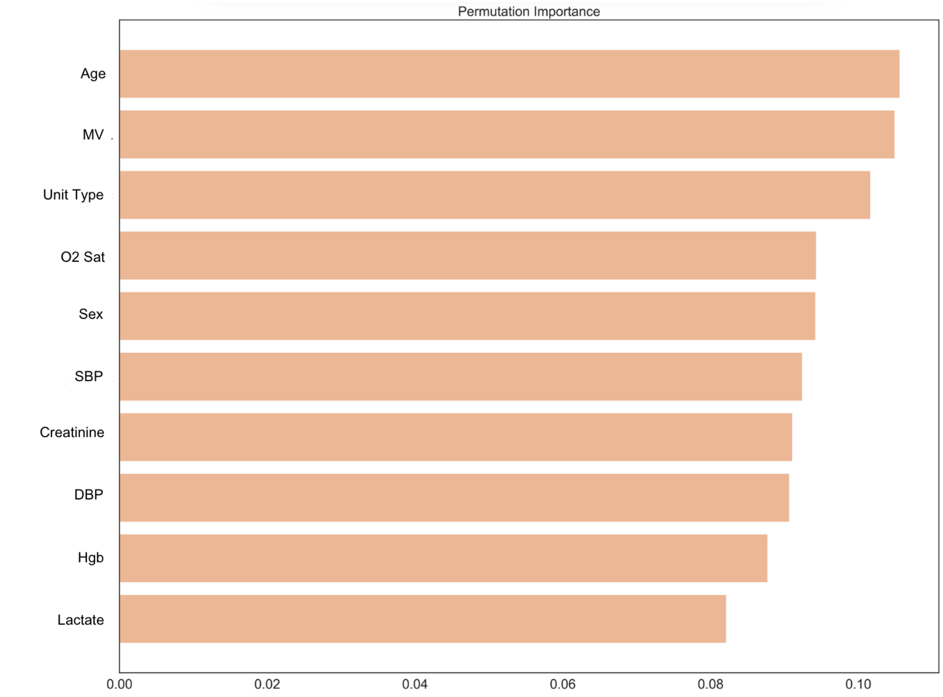

(b)

Fig. 3: The feature importance of the top 10 features for survival prediction on a) MIMIC-IV and b) eICU test sets evaluated over 10 iterations.

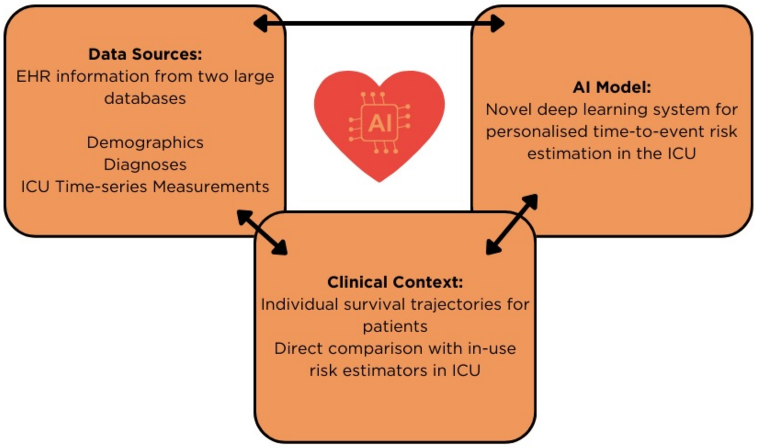

Fig. 4: Proposed framework incorporating interdisciplinary areas of clinical intensive care and applied machine learning to dynamic risk estimation

REFERENCES

[1] Wiegrebe S, Kopper P, Sonabend R, Bischl B, Bender A. Deep learning for survival analysis: a review. Artificial Intelligence Review. 2024;57(3):65.

[2] Kvamme H, Borgan Ø. Continuous and discrete-time survival prediction with neural networks. arXiv preprint arXiv:191006724. 2019.

[3] Yu CN, Greiner R, Lin HC, Baracos V. Learning patient-specific cancer survival distributions as a sequence of dependent regressors. Advances in neural information processing systems. 2011;24.

[4] Fotso S. Deep neural networks for survival analysis based on a multi-task framework. arXiv preprint arXiv:180105512. 2018.

[5] Kvamme H, Borgan Ø. The brier score under administrative censoring: Problems and solutions. arXiv preprint arXiv:191208581. 2019.

[6] Lee C, Zame W, Yoon J, Van Der Schaar M. Deephit: A deep learning approach to survival analysis with competing risks. In: Proceedings of the AAAI conference on artificial intelligence. vol. 32; 2018. .

[7] Ren K, Qin J, Zheng L, Yang Z, Zhang W, Qiu L, et al. Deep recurrent survival analysis. In: Proceedings of the AAAI Conference on Artificial Intelligence. vol. 33; 2019. p. 4798-805.

[8] Sun Z, Dong W, Shi J, He K, Huang Z. Attention-based deep recurrent model for survival prediction. ACM Transactions on Computing for Healthcare. 2021;2(4):1-18.

[9] Nagpal C, Li X, Dubrawski A. Deep survival machines: Fully parametric survival regression and representation learning for censored data with competing risks. IEEE Journal of Biomedical and Health Informatics. 2021;25(8):3163-75.

[10] Gensheimer MF, Narasimhan B. A scalable discrete-time survival model for neural networks. PeerJ. 2019;7:e6257.

[11] Kvamme H, Borgan Ø, Scheel I. Time-to-event prediction with neural networks and Cox regression. arXiv preprint arXiv:190700825. 2019.

[12] Katzman JL, Shaham U, Cloninger A, Bates J, Jiang T, Kluger Y. DeepSurv: personalized treatment recommender system using a Cox proportional hazards deep neural network. BMC medical research methodology. 2018;18(1):1-12.

[13] Thammasorn P, Schaub SK, Hippe DS, Spraker MB, Peeken JC, Wootton LS, et al. Regularizing the Deepsurv network using projection loss for medical risk assessment. IEEE Access. 2022;10:8005-20.

[14] Friedman M. Piecewise exponential models for survival data with covariates. The Annals of Statistics. 1982;10(1):101-13.

[15] Hochreiter S, Schmidhuber J. Long short-term memory. Neural computation. 1997;9(8):1735-80.

[16] Staudemeyer RC, Morris ER. Understanding LSTM–a tutorial into long short-term memory recurrent neural networks. arXiv preprint arXiv:190909586. 2019.

[17] Understanding LSTM Networks – colah's blog — colah.github.io;. [Accessed 20-07-2024]. https://colah.github.io/posts/2015-08-Understanding-LSTMs/.

[18] Lipes J, Mardini L, Jayaraman D. Sex and mortality of hospitalized adults after admission to an intensive care unit. American Journal of Critical Care. 2013;22(4):314-9.

[19] Wang J, Li N, Mu Y, Wang K, Feng G. Association between serum albumin creatinine ratio and all-cause mortality in intensive care unit patients with heart failure. Frontiers in Cardiovascular Medicine. 2024;11:1406294.

[20] Zhang Z, Xu X, Ni H, Deng H. Platelet indices are novel predictors of hospital mortality in intensive care unit patients. Journal of critical care. 2014;29(5):885-e1.

[21] Schuurmans J, van Rossem BT, Rellum SR, Tol J, Kurucz VC, van Mourik N, et al. Hypotension during intensive care stay and mortality and morbidity: a systematic review and meta-analysis. Intensive care medicine. 2024:1-10.

TABLE V: Features extracted from the MIMIC-IV database. The features include demographic data collected for all patients, ICU unit-specific information like the type of unit, hospital information like regional location, time since admission, vital signs including respiratory rate and blood pressure, and biochemical measurements including blood glucose and haemoglobin.

**Static Variables**			
Feature	*Type*	*Feature*	*Type*
Sex	binary	Motor Response	continuous
Age	integer	Verbal Response	continuous
Height	continuous	Ethnicity	categorical
Weight	continuous	Unit Type	categorical
Hour of Admission	integer	Admission Location	categorical
Time Since Admission	continuous		
**Time-series Variables**			
Feature	*Type*	*Feature*	*Type*
		GCS - Eye	continuous
Eye Response	continuous	GCS - Motor	continuous
Braden Score	continuous	GCS - Verbal	continuous
Strength L Arm	continuous	Daily Weight	continuous
Strength R Arm	continuous	ALT	continuous
Strength L Leg	continuous	AST	continuous
Strength R Leg	continuous	HCO3	continuous
Insurance	categorical	Hct	continuous
ALT	continuous	Alkaline Phosphatase	continuous
Anion Gap	continuous	AST	continuous
Base Excess	continuous	Bicarbonate	continuous
Bilirubin	continuous	Calcium	continuous
Total CO2	continuous	Chloride	continuous
Creatinine	continuous	Glucose	continuous
Hematocrit	continuous	Hemoglobin	continuous
INR(PT)	continuous	Lactate	continuous
MCH	continuous	MCHC	continuous
MCV	continuous	Magnesium	continuous
PT	continuous	PTT	continuous
Phosphate	continuous	Platelet Count	continuous
Potassium	continuous	RDW	continuous
Red Blood Cells	continuous	Sodium	continuous
Urea Nitrogen	continuous	White Blood Cells	continuous
pCO2	continuous	pH	continuous
pO2	continuous	JH-HLM	continuous
Dyspnea Assessment	continuous	Daily Weight	continuous
Glucose	continuous	Heart Rate	continuous
DBP	continuous	SBP	continuous
O2 Flow	continuous	O2 Sat (%)	continuous
Pain Level	continuous	Pain Level Response	continuous
Phosphorous	continuous	Respiratory Rate	continuous
Richmond-RAS Scale	continuous	Temperature (°F)	continuous

TABLE VI: Features extracted from the eICU database. The features include demographic data collected for all patients, ICU unit-specific information like type and number of beds, hospital information like regional location and teaching status, vital signs including respiratory rate and blood pressure, and biochemical measurements including troponin and levels of potassium and protein in the blood.

**Feature**	**Type**	**Feature**	**Type**
Sex	binary	Unit Stay Type	categorical
Age	integer	Num Beds Category	categorical
Height	continuous	Region	categorical
Weight	continuous	Teaching Status	binary
Ethnicity	categorical	Physician Speciality	categorical
Unit Type	categorical	Unit Type	categorical
Unit Admit Source	categorical	Mechanical Ventilation	binary
Unit Visit Number	categorical		
**Time-series Variables**			
Feature	*Type*	*Feature*	*Type*
		Base Excess	continuous
-basos	continuous	FiO2	continuous
-eos	continuous	HCO3	continuous
-monos	continuous	Hct	continuous
-polys	continuous	Hgb	continuous
ALT	continuous	MCH	continuous
AST	continuous	MCHC	continuous
BUN	continuous	MCV	continuous
O2 Sat (%)	continuous	MPV	continuous
PT-INR	continuous	PT	continuous
RBC	continuous	PTT	continuous
RDW	continuous	WBC	continuous
Alkaline ph.	continuous	Albumin	continuous
Bedside Glucose	continuous	Anion Gap	continuous
Calcium	continuous	Bicarbonate	continuous
Creatinine	continuous	Glucose	continuous
Lactate	continuous	Magnesium	continuous
pH	continuous	paCO2	continuous
paO2	continuous	Phosphate	continuous
Platelets	continuous	Potassium	continuous
Sodium	continuous	Bilirubin	continuous
Protein	continuous	Troponin - I	continuous
Urinary s. Gravity	continuous	mean BP	continuous
SBP	continuous	DBP	continuous

TABLE VII: Ablation study experiments with DySurv on MIMIC-IV using time-series and static components with the optimal hyperparameter combination. The top strategy was kept as the optimal one when adding lower strategies. For example, once the inclusion of dropout was found to contribute positively to the results, it was included in the model architecture, then the addition of batch normalisation was tested.

	C_{ind}^{td}	IBS	IBLL
**Dropout** (p **= 0.5**)			
With	57.9	0.122	0.320
Without	53.1	0.145	0.389
**Batch Normalisation**			
With	57.6	0.130	0.322
Without	57.9	0.122	0.320
**Conditional term concatenation**			
With	57.9	0.122	0.320
Without	55.7	0.144	0.377
DySurv (+ time-series)	57.9	0.122	0.320
